# Spatial heterogeneity of low-birthweight deliveries on the Kenyan coast

**DOI:** 10.1186/s12884-023-05586-6

**Published:** 2023-04-19

**Authors:** Moses M. Musau, Stella Mwakio, David Amadi, Amek Nyaguara, Philip Bejon, James A. Berkley, Robert W. Snow, Alice Kamau

**Affiliations:** 1 Population & Health Unit, Kenya Medical Research Institute (KEMRI) - Wellcome Trust Research Programme, Nairobi, Kenya; 2 Kenya Medical Research Institute (KEMRI) - Wellcome Trust Research Programme, Kilifi, Kenya; 3 Centre for Tropical Medicine and Global Health, Nuffield Department of Clinical Medicine, University of Oxford, Oxford, UK; 4 Centre for Clinical Vaccinology and Tropical Medicine, Churchill Hospital, University of Oxford, Oxford, UK

**Keywords:** Low birthweight, Spatial heterogeneity, Accessibility, Kilifi, Kenya

## Abstract

**Background:**

Understanding spatial variations in health outcomes is a fundamental component in the design of effective, efficient public health strategies. Here we analyse the spatial heterogeneity of low birthweight (LBW) hospital deliveries from a demographic surveillance site on the Kenyan coast.

**Methods:**

A secondary data analysis on singleton livebirths that occurred between 2011 and 2021 within the rural areas of the Kilifi Health and demographic surveillance system (KHDSS) was undertaken. Individual-level data was aggregated at enumeration zone (EZ) and sub-location level to estimate the incidence of LBW adjusted for accessibility index using the Gravity model. Finally, spatial variations in LBW were assessed using Martin Kulldorf’s spatial scan statistic under Discrete Poisson distribution.

**Results:**

Access adjusted LBW incidence was estimated as 87 per 1,000 person years in the under 1 population (95% CI: 80, 97) at the sub-location level similar to EZ. The adjusted incidence ranged from 35 to 159 per 1,000 person years in the under 1 population at sub-location level. There were six significant clusters identified at sub-location level and 17 at EZ level using the spatial scan statistic.

**Conclusions:**

LBW is a significant health risk on the Kenya coast, possibly under-estimated from previous health information systems, and the risk of LBW is not homogenously distributed across areas served by the County hospital.

**Supplementary Information:**

The online version contains supplementary material available at 10.1186/s12884-023-05586-6.

## Background

The World Health Organization (WHO) defines newborns weighing less than 2500 g, irrespective of gestational age, as low birthweight (LBW) [1]. LBW is considered a crucial determinant of infant mortality, particularly in the first month of life [2, 3]. Those who survive are more likely to suffer from stunted growth [4, 5], poor cognitive development [6] and long-term consequences extending to adulthood [7].

The United Nations estimates that of the 20.5 million LBW babies (representing ~ 14% of all live births), nearly 25% occurred in the African region in 2015 [8, 9]. In 2015, the number of LBW among live births was estimated to have increased from 4.4 million in 2000 to 5 million in sub-Saharan Africa (SSA) [10]; a figure likely to be under-estimated as many deliveries occur at home or at primary care clinics where official figures are not reported [11].

Reducing LBW has long been recognized as a public health priority and it is now a global commitment with the adoption of the Global Nutrition Targets in 2012. The WHO has a goal to reduce LBW by 30% by 2025 [12]. However, progress remains inadequate especially in low- and middle-income countries (LMIC). LBW not only reflects the health of the child at birth but equally reflects the health status of the mother during pregnancy, including malnutrition, malaria, other infections, and poor pregnancy-related health service utilization [13]. Some of these factors are modifiable through early and comprehensive prenatal care and these factors have been shown to vary across broad regional scales [14].

Understanding spatial variations in health outcomes is a fundamental component in the design of effective, efficient public health strategies. The identification of areas with high disease burden enables national and local health authorities to target limited resources to maximise impact and understand the finer scale epidemiology of poor health. There have been very few investigations of the national spatial variations in LBW burden [14–18] or finer spatial scales below national levels [15–18]. In Kenya, LBW rates significantly varied sub-nationally [19] and similar findings have been reported in Ethiopia [20], Namibia [21], India [14, 22], Indonesia [23] and in developed nations such the USA [16–18].

Global maternal and newborn health initiatives, Every Newborn Action Plan and Ending Preventable Maternal Mortality, have identified priority indicators derived from facility-based data as important [24, 25]. However, there have been very few facility-based data investigating the spatial patterns of LBW in SSA. In addition, far fewer studies have investigated the potential of facility-based data to identify LBW hotspots with studies in the African region relying on data from demographic health surveys (DHS) [20, 21]. Here we analyse the spatial heterogeneity of LBW hospital deliveries from a demographic surveillance site on the Kenyan coast.

## Methods

### Study area and context

This study was a retrospective analysis of routine data collected at Kilifi County Hospital [26, 27], matched to the Kilifi Health and Demographic Surveillance System (KHDSS) [28]. The KHDSS is located on the Kenyan Coast and subdivided into 37 sub-locations (an administrative unit covering ~ 39,000 people), which are further subdivided into 186 enumeration zones (EZ) each consisting of approximately 226 homesteads [28]. Kilifi County hospital (KCH), located in Kilifi township, is the referral hospital for the population in this area and has a maternity ward that attends to pregnant mothers seeking antenatal care and delivery services that records ~ 4,000 deliveries each year [26]. For the purposes of the present study the area was restricted to the rural extent which represents ~ 80% of the KHDSS population. The population in the urban part of KHDSS is mostly transient; people tend to stay for short periods including those accessing KCH before proceeding to their permanent residence and were excluded from the present analysis (Additional file 1: Figure S1; grey regions).

### Data collection

Data was obtained from the maternity ward surveillance system at KCH established in January 2011 to standardize maternal admission procedures and improve standard of care under the Kilifi Perinatal and Maternity research (KIPMAT) study [26]. Registration of all admissions for mothers who present in labour is undertaken by trained fieldworkers present at the maternity ward 24 h, seven days a week [26, 27]. Clinical assessment and documentation were undertaken by nursing and medical staff as part of routine care using a structured maternal admission record form. The study routinely collected information on the residential address of the mother and the newborn outcomes i.e. birth status (live birth or stillbirth) and birth weight. Weight was measured within the first hour of birth using a balanced Seca 354 digital baby scale manufactured by Seca GmbH & Co. KG, Germany. The residential details of each mother were linked to the KHDSS enumeration zone (EZ). All data were captured using a customised tool built on a PHP web-based interface and data saved onto MySQL database.

### Statistical analysis

#### Data inclusion and exclusions

**Fig. 1:**
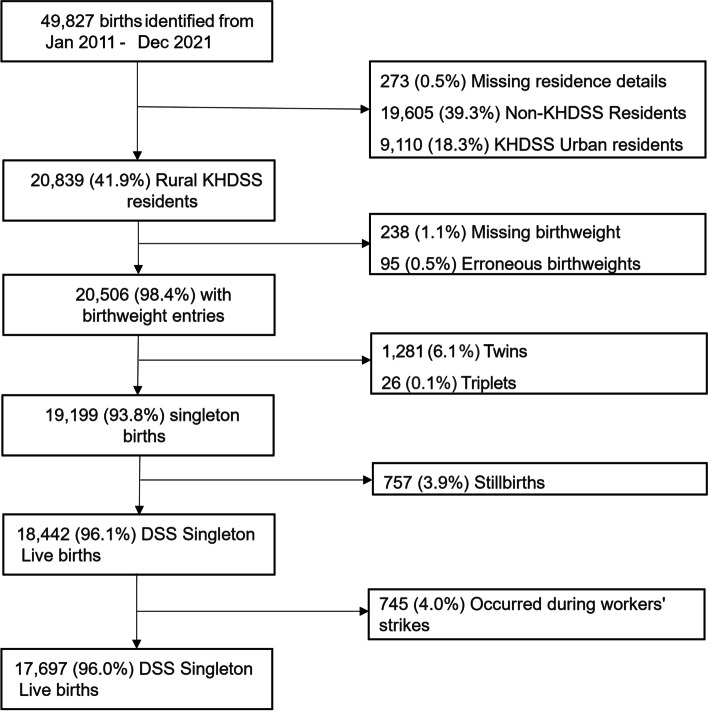
The inclusion and exclusion criteria for deliveries as KCH. The population in the Kilifi Urban area is characterised by temporary (transient) residents thus presenting a challenge in establishing whether the permanent residence of this population is within the KHDSS and was thus excluded. In comparison to singleton births, multiple births are associated with increased odds of LBW [32]. Therefore, multiple births were excluded to remove bias towards LBW outcome. Still born babies have increased chances of being born with LBW [33]. Therefore, their inclusion would introduce bias in the data. Health workers’ strikes occurred in Mar 2012, Dec 2012, Dec-2016 to Feb-2017, Jun-2017 to Nov-2017, Dec-2020 to Mar-2021

This study included deliveries between January 2011 and December 2021. Data were incomplete during health workers strikes which occurred in 2016, 2017, 2020 and 2021 [29–31] and months with incomplete data that coincided with the health workers’ strikes were excluded. All still born babies were excluded. In addition, all multiple births were excluded, that have intrinsically increased odds of LBW [27, 32]. Finally, newborns with missing or erroneous birth weight records were also excluded from the analysis (Fig. 1). The analysis therefore focused on singleton livebirths that occurred during 117 months of surveillance between 2011 and 2021 within the rural areas of the KHDSS.

#### Overview of data analysis

The completeness of disease events described at health facilities will be dependent on a) accessibility to the recording facility, for example those with poorer access are less likely to be documented [11, 34, 35], b) higher at-risk population densities will generate more events assuming risk is homogenous, and c) illness severity, more complicated disease events are more likely to travel further [35, 36]. We therefore adjusted “incidence” based on likely density of births during the interval (estimated from census populations of resident infants) and a measure of accessibility to the KCH maternity ward (estimated as travel time to the hospital).

#### Low birthweight (LBW) incidence

The individual-level data was aggregated at EZ as the smallest unit of spatial analysis, equivalent to village, to estimate the incidence of LBW. The yearly population counts of children under 1 year for these EZs were derived from actual continuous population surveillance data in the KHDSS. The person years of observation (PYO) were adjusted to only include months when strikes did not affect admissions to the maternity ward, hereafter referred to as weighted population for children under 1 year. The overall incidence rate of LBW was computed using exact Poisson distribution with the 95% confidence intervals (CI) obtained from 1,000 bootstrap replicates, with resampling at the EZ level. For each EZ, the incidence rate of LBW was computed as follows:

$${LBW}_{i}=\frac{{LBWcount}_{i}}{{PYO}_{i}}\times \mathrm{1,000}$$

where LBW count_i_ are the raw counts of LBW cases at EZ_i_, PYO_i_ is the weighted population for children under 1 year for EZ_i_ and LBW_i_ is the LBW incidence per 1,000 person years in the under 1 population for EZ_i_. These incidence estimates, however, represent a minimum measure of the true community burden of LBW, as LBW events may have occurred outside of Kilifi County hospital. This was repeated for a lower spatial resolution, sub-location, which represents a wider local administrative area sometimes used by county governments.

#### Adjusting LBW incidence for access

To account for variable geographical access to health care, an accessibility index was computed using the Gravity model [37, 38] which attempts to represent the potential interaction between the population and health care service providers such that the likelihood of interaction decreases with increasing travel impedance, described in detail in the Additional file 2 [34, 39]. Briefly, travel impedance was represented by the time taken to travel to KCH from the EZs. To calculate travel time from each EZ, a friction surface accounting for physical barriers, digital elevation model, different landcover types, roads and the associated walking and motorized speeds was used in AccessMod 5.6.0. The travel times obtained were then input in the Gravity model to obtain the spatial accessibility index for each EZ. To compute the adjusted LBW incidence, the benchmark multiplier method [39] which corrects for the possible underestimation of LBW by multiplying the observed cases with the inverse probability of detecting a case based on spatial accessibility index was used. The adjusted LBW cases was computed as:

$${LBW}_{adj,i}=\frac{{LBWcount}_{i}}{{A}_{i}}$$

where LBW count_i_ are the raw counts of LBW cases at EZ_i_, A_i_ is the access index for EZ_i_ and LBW_adj,i_ is the adjusted LBW counts for EZ_i._ The adjusted LBW counts at EZ level were aggregated to sub-location level to compute the incidence of LBW. Spearman’s rank correlation coefficient was used to assess whether the bias in the estimation of LBW incidence due to geographic access to KCH was present after adjustment.

#### Local spatial cluster detection

To assess whether there were spatial variations in LBW, spatial heterogeneity tests were performed on the adjusted LBW counts using Martin Kulldorf’s spatial scan statistic estimated in SaTScan software [40]. Spatial clusters were defined as geographical areas experiencing significantly more LBW cases than would be expected by chance. SaTScan imposes a circular scanning window that moves across the study area with radius varying from zero to a maximum of 50% (prespecified by the user) of the population in the sampling frame or in terms of geographical size using the circle radius. The log-likelihood ratio test is performed for each scanning window by comparing the number of cases inside the window to those outside to derive a log likelihood statistic [40]. To test the null hypothesis of complete spatial randomness, SaTScan employs Monte Carlo simulations where for each simulation run, the observed cases are randomly permuted in space across the entire set of data locations. Monte Carlo simulated and observed log-likelihood ratios are then compared to determine statistical significance of the most likely clusters accounting for multiple testing for the variations in window location and radius.

To detect hotspots of LBW cases, Kulldorf’s spatial scan statistic under the Discrete Poisson distribution was used. The goal was to investigate the existence of spatial heterogeneity at the EZ and sub-location levels, therefore, the maximum scanning radius was set to 1 km to allow each unit to be assessed as a possible standalone cluster. The inputs for SaTScan were adjusted LBW counts as cases, under 1 year population as the population at risk and EZ or sub-location centroids as the centres of the scanning windows. The most likely cluster was identified based on the maximum log likelihood ratio (LLR) and other clusters with statistically significant log likelihood values were defined as secondary clusters.

Previously, socioeconomic factors have been shown to be associated with the odds of LBW [20]. The smallest social unit which are likely to share socioeconomic characteristics located at the lowest governmental community policing structure in Kenya comprises of ten nearest neighbouring households [41]. In our data, the maximum distance for a homestead to have ten neighbours was 2.3 km, therefore, as a sensitivity analysis, the maximum scanning radius was altered at 2 km and 2.3 km.

Martin Kulldorf’s spatial scan statistic (SaTScan) was performed using R version 4.1.0 under the rsatscan package. The resulting cluster shapefiles were created using R version 4.1.0 under the rgdal package. Visualisation of maps and clusters was done using ArcGIS version 10.5.

## Results

### Data description

There were 49,827 births delivered at KCH between 2011 and 2021. Of these deliveries, we excluded 273 (0.5%) with missing residence details, 19,605 (39.3%) and 9,110 (18.3%) residing outside the KHDSS and in the KHDSS urban extent, respectively (Fig. 1). In addition, we excluded 333 (1.6%) deliveries with either missing or anomalous birthweight and 1,307 (7.9%) multiple births (1,281 twins and 26 triplets). Of the remaining 19,199 births, 757 (3.9%) were still births and 745 (4.0%) occurred during health workers’ strikes and were excluded (Fig. 1). A total of 17,697 live singleton births were included in the analysis and the median number of live births at EZ level was 82 ranging between 5 and 942 and the median under 1 population at EZ level was 504 and ranged between 69 and 1445. The distribution of deliveries at KCH and the weighted under 1 population followed a similar pattern in the EZ and sub-location levels (Additional file 1; Figure S1).

### LBW incidence

**Fig. 2:**
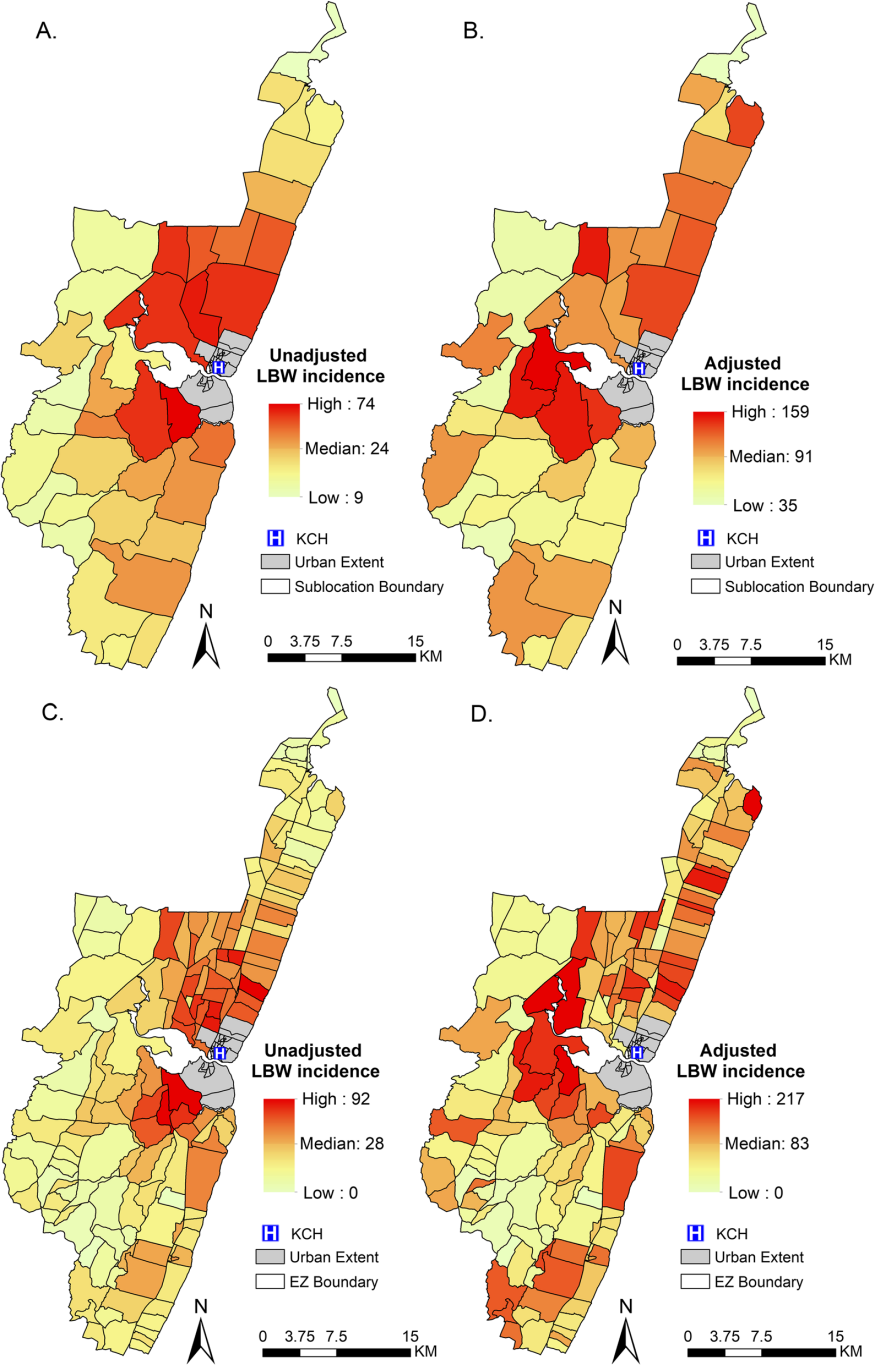
The distribution of the unadjusted and adjusted LBW incidence with the red shade showing higher incidence and yellow shade showing lower incidence. The unadjusted incidence represents the LBW events that occurred at KCH while the adjusted incidence represents LBW events that have been corrected for underestimation due to variable geographical access to health care. **A** the distribution of unadjusted LBW incidence at sub-location level. **B** distribution of adjusted LBW incidence at sub-location level. **C** distribution of unadjusted LBW incidence at EZ level. **D** distribution of adjusted LBW incidence at EZ level

Of all the observed deliveries at KCH, 15% (2,667/17,697) were of LBW. The unadjusted LBW incidence at sub-location was estimated as 32 per 1,000 person years in the under 1 population (95% CI: 26, 37); a rate similar to that described at the EZ level. There were variations in the unadjusted LBW incidence across the study area with higher rates of LBW found in areas proximal to KCH (Fig. 2A & C). At sub-location level, the unadjusted incidence of LBW varied from as low as 9 LBW cases per 1,000 person years in the under 1 population to as high as 74 per 1,000 person years in the under 1 population (Fig. 2A). There was a clear distance decay in the unadjusted LBW incidence at the sub-location level (*r* = -0.757: *p*-value < 0.001, Additional file 3; Figure S4A) but when adjusted for accessibility index, there was no association between adjusted LBW incidence and travel time (*r* = -0.003: *p*-value = 0.986 Additional file 3; Figure S4B). A similar pattern was seen when EZ level data was used (Additional file 3; Figure S4C & D). After adjusting for accessibility index, we estimate that the number of LBW cases identified at KCH represented only 35.9% (2,667/7,439) of the total possible number of LBW cases. The overall adjusted LBW incidence was estimated as 87 per 1,000 person years in the under 1 population (95% CI: 80, 97) at the sub-location level again similar to EZ. The adjusted incidence ranged from 35 to 159 per 1,000 person years in the under 1 population at sub-location level (Fig. 2B). There were differences in spatial variations between the unadjusted and adjusted LBW incidence at both sub-location and EZ levels (Fig. 2).

### Spatial patterns of LBW incidence

**Fig. 3:**
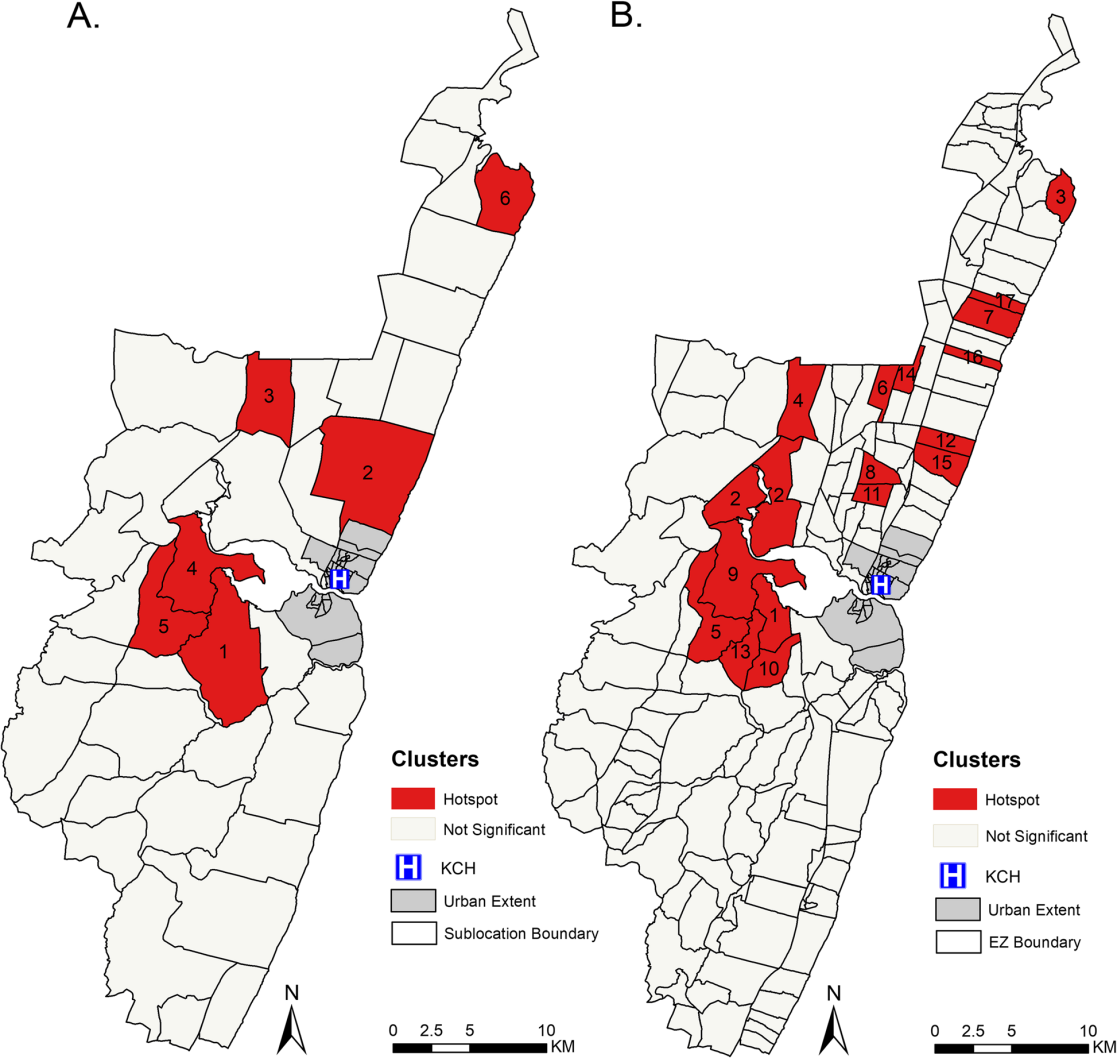
The distribution of significant high incidence clusters (hotspots) identified using the SaTScan software. **A** shows clusters obtained at sub-location level with 1 representing the primary clusters and 2 – 6 are the secondary clusters. **B** shows clusters obtained at EZ level where 1 indicates the primary clusters and 2 – 17 are the secondary clusters

To assess whether these spatial variations of LBW were higher than would be expected by random chance, the adjusted LBW data was used in the SaTScan analysis using the Poisson spatial model. The clusters are shown on the maps by sub-locations and EZs levels (Fig. 3). The spatial distribution in the adjusted data revealed six clusters (Fig. 3A; Table 1). The primary cluster was in the central area of the KHDSS (Fig. 3A; cluster 1) and had an average incidence of 138 per 1,000-person years with 1.6 (*p* < 0.001) times higher risk of being LBW compared to areas outside the window. Within the other five significantly high incidence spatial clusters identified (Fig. 3A), the risk of LBW was between 1.31 and 1.79 times higher than those outside the windows (Table 1). Variations in the size of the maximum spanning radius yielded similar results (Additional file 4; Figure S5A & B).

**Table 1:** Spatial clusters (hotspots) of LBW detected by SaTScan at sub-location level, ordered from the cluster with the highest LLR

Cluster Number	Adjusted LBW Counts	Expected Counts	Relative Risk	*P* value
Hotspot				
1	595	382.58	1.60	< 0.001
2	699	546.40	1.31	< 0.001
3	194	127.65	1.53	< 0.001
4	92	51.62	1.79	< 0.001
5	107	68.02	1.58	< 0.001
6	236	178.86	1.33	0.002

At the EZ level, 17 clusters were identified (Fig. 3B). The primary cluster was located in the central region (Fig. 3B: cluster 1) in a similar location to that under sub-location level. The primary hotspot had an average incidence of 130 per 1,000-person years with 2.29 (*p* < 0.001) times higher risk of being LBW compared to areas outside the window (Table 2). There were 16 other significant clusters identified and the relative risk ranged between 1.41 and 2.46 (Table 2). Comparing clusters identified at both levels, clusters in the EZ level were in similar positions as those identified at sub-location level (Fig. 3). When we varied the radius of the cluster at EZ level, the results were similar (Additional file 4; Figure S5C & D), for the entire study period (2011 – 2021).

**Table 2:** Spatial clusters (hotspots) of LBW detected by SaTScan at EZ level, ordered from the cluster with the highest LLR

Cluster Number	Adjusted LBW Count	Expected Count	Relative Risk	*P* value
Hotspot				
1	183	81.19	2.29	< 0.001
2	137	59.26	2.34	< 0.001
3	101	41.42	2.46	< 0.001
4	141	80.37	1.77	< 0.001
5	75	35.29	2.14	< 0.001
6	99	52.90	1.88	< 0.001
7	87	44.57	1.96	< 0.001
8	97	53.43	1.83	< 0.001
9	92	51.61	1.79	< 0.001
10	132	85.05	1.56	< 0.001
11	70	39.98	1.76	0.002
12	109	71.19	1.54	0.004
13	98	63.50	1.55	0.010
14	53	30.29	1.75	0.024
15	74	46.51	1.60	0.025
16	134	95.76	1.41	0.027
17	42	22.55	1.87	0.036

## Discussion

Information on the fine spatial patterns of LBW is rare in SSA. We have explored the incidence of LBW among deliveries within a discrete community on the Kenyan coast, accounting for expected deliveries and their likelihood of accessing hospital services at delivery using the Kulldorf’s spatial scan statistic under the Discrete Poisson distribution. Access adjusted LBW incidence was estimated to be 87 per 1,000 person years in the under 1 population. A recent study conducted in Kenya at sub-county level (a lower administrative unit) reported LBW incidence of 45 per 1000 live births [19], however, this study used incomplete routine health service data from national reporting systems. Importantly, we found a marked variability of LBW incidence at finer spatial resolutions, below the county and sub-county levels described by Odhiambo and Sartorius (2021) [19]. We identified six clusters at sub-location level and 17 at EZ level. The spatial heterogeneity of LBW has been described sub-nationally in SSA previously [19–21] but not within sub-national administrative areas.

The direct and indirect causes of LBW are manifold and there are spatial variations in maternal exposure to infectious diseases, notably malaria, nutrition and access to antenatal care (ANC) during pregnancy. Across Kenya, these risk factors all vary significantly [42, 43], potentially contributing to the national variation in LBW incidence [19]. It is reasonable to assume that these same determinants of LBW define risks at higher spatial resolutions and potentially influences the clustering we observed within the KHDSS. Our intention was to describe heterogeneity, adjusting for accessibility, rather than explore reasons for clustering. However, it is notable that adjusted incidence (Fig. 2A and B) and specific, statistically significant clustering of LBW (Fig. 3b) occurred predominantly in areas north of the hospital compared to areas south of the hospital. Northern areas have much lower malaria exposure and disease incidence compared to southern areas [44]. As the intensity of malaria transmission declines, rarer parasite exposures during pregnancy may result in poorer birth outcomes. While speculative this would require further analysis alongside other potential factors related to community access to ANC, socio-economic status and food security, beyond the scope of the present paper.

It is important to note that 1) LBW incidence was only adjusted for spatial access to KCH as measures of spatial access are effective in understanding how likely a population is to utilize the available healthcare services as previously described [37]. However, if aspatial factors such as affordability or health belief systems among other aspatial factors significantly influence utilization of healthcare services, this could have led to the overestimation or underestimation of the true incidence and further adjustments based on such factors should be made – a limitation to the current study as such data was not available. 2) The period of consideration for the current study was long (2011 to 2021). When temporal trends of LBW clusters were analysed (results not shown), the spatial clusters jumped both spatially and temporarily because of the small numbers included when data was disaggregated annually – also a limitation of this study. To detect geographical clustering of LBW SaTScan was used, however, constructing confidence intervals for the relative risk remains an open challenge and a formal method for constructing confidence interval is needed. Although a sensitivity analysis was conducted, the generalizability of these results will be reinforced if similar studies are conducted in other populations.

Our results suggest that birth outcomes are related to geographic location. Clustering of disease outcomes suggests a more intensive investigation of the spatial risks associated with LBW that may inform a more targeted approach to maternal and newborn interventions (policy/care package/services). As health systems and community care become more tailored and sophisticated at county levels, using local epidemiology data to guide intervention becomes more important. LBW is a significant health risk on the Kenya coast, possibly under-estimated from previous health information systems, and the risk of LBW is not homogenously distributed across areas served by the County hospital.

## Supplementary Information

**Additional file 1: Fig. S1.** The distribution of observed deliveries and weighted under 1 population. The darker the shade the higher the value. Panels A and B: shows the distribution at sub-location level. Panels C and D: shows the distribution at EZ level, respectively. The number of deliveries and weighted under 1 population on the maps are for the entire study period (2011 – 2021).

**Additional file 2: Table S1.** Speeds assigned to different road classes and land covers.** Figure S2. **Distribution of travel time from EZ centroids to KCH; the darker the shade the longer the travel time.** Figure S3.** Distribution of access index from EZ centroids to KCH; the darker the shade the higher the access index.

**Additional file 3: Figure S4.** The association between LBW incidence and travel time. Panels A and C: shows the distance decay for unadjusted LBW incidence at sub-location and EZ level, respectively. Panels B and D: shows the distance decay for adjusted LBW incidence at sub-location and EZ level, respectively.

**Additional file 4: Figure S5.** Sensitivity analysis using varying maximum scanning radius for cluster identification using SaTScan software. Panel A and B: The radius was set at 2 km and 2.3 km at sub-location level, respectively. Panel C and D: The radius was set at 2 km and 2.3 km at EZ level, respectively. The clusters identified were in similar locations as those reported using the 1 km radius for the entire study period (2011 – 2021).

## Data Availability

Data that support the findings of this study are available from the KEMRI Institutional Data Access/Ethics Committee. Details of the guideline can be found in the KEMRI-Wellcome data sharing guidelines (https://kemri-wellcome.org/about-us/#ChildVerticalTab_15). Access to data is provided via the KEMRI Wellcome Data Governance Committee: dgc@kemri-wellcome.org through Marianne Munene (mmunene@kemri-wellcome.org).

## Abbreviations

ANC: Antenatal Care
CI: Confidence Interval
EZ: Enumeration Zone
KCH: Kilifi County Hospital
KHDSS: Kilifi Health and Demographic Surveillance System
KIPMAT: Kilifi Perinatal and Maternity Research
LBW: Low Birthweight
LLR: Log Likelihood Reatio
LMIC: Low- and Middle-Income Countries
PYO: Person Years of Observation
SSA: Sub-Saharan Africa
UNICEF: United Nations Children’s Fund
USA: United States of America
WHO: World Health Organization

## References

[1] WHO. International statistical classification of diseases and related health problems. World Health Organization; 2004. https://apps.who.int/iris/handle/10665/42980. Accessed 28 Dec 2022.

[2] Stewart AL, Reynolds EOR, Lipscomb AP (1981). outcome for infants of very low birthweight: survey of world literature. The Lancet.

[3] Lawn JE, Cousens S, Zupan J (2005). Lancet Neonatal Survival Steering Team. 4 million neonatal deaths: when? Where? Why?. Lancet.

[4] Beck GJ, van den Berg BJ (1975). The relationship of the rate of intrauterine growth of low-birth-weight infants to later growth. J Pediatr.

[5] Christian P, Lee SE, Angel MD, Adair LS, Arifeen SE, Ashorn P (2013). Risk of childhood undernutrition related to small-for-gestational age and preterm birth in low- and middle-income countries. Int J Epidemiol.

[6] Goldenberg RL, Hoffman HJ, Cliver SP (1998). Neurodevelopmental outcome of small-for-gestational-age infants. Eur J Clin Nutr.

[7] Jornayvaz FR, Vollenweider P, Bochud M, Mooser V, Waeber G, Marques-Vidal P (2016). Low birth weight leads to obesity, diabetes and increased leptin levels in adults: The CoLaus study. Cardiovasc Diabetol.

[8] UNICEF-WHO. Low birthweight estimates: levels and trends 2000–2015. Geneva: World Health Organization; 2019. https://www.unicef.org/reports/UNICEF-WHO-low-birthweight-estimates-2019. Accessed 08 Jan 2023

[9] WHO. WHO recommendations for care of the preterm or low birth weight infant. Geneva: World Health Organization; 2022. https://reliefweb.int/report/world/who-recommendations-care-preterm-or-low-birth-weight-infant. Accessed 19 Jan 2023. 36449655

[10] Blencowe H, Krasevec J, de Onis M, Black RE, An X, Stevens GA (2019). National, regional, and worldwide estimates of low birthweight in 2015, with trends from 2000: a systematic analysis. Lancet Glob Health.

[11] Gabrysch S, Campbell OMR (2009). Still too far to walk: Literature review of the determinants of delivery service use. BMC Pregnancy Childbirth.

[12] WHO. Global nutrition targets 2025: low birth weight policy brief. Geneva: World Health Organization; 2014. https://www.who.int/publications/i/item/WHO-NMH-NHD-14.5. Accessed 06 Jan 2023.

[13] Menéndez C, D’Alessandro U, ter Kuile FO (2007). Reducing the burden of malaria in pregnancy by preventive strategies. Lancet Infect Dis.

[14] Banerjee A, Singh AK, Chaurasia H (2020). An exploratory spatial analysis of low birth weight and its determinants in India. Clin Epidemiol Glob Health.

[15] Rip MR, Keen CS, Woods DL (1987). Spatial variations of low birthweight in Cape Town. J Trop Pediatr..

[16] Tu W, Tedders S, Tian J (2012). An exploratory spatial data analysis of low birth weight prevalence in Georgia. Appl Geogr.

[17] Tian J, Tu W, Tedders S, Chen D (2013). A spatial-temporal analysis of low birth weight prevalence in Georgia. USA GeoJournal.

[18] Burns JJ, Livingston R, Amin R (2020). The proximity of spatial clusters of low birth weight and risk factors: defining a neighborhood for focused interventions. Matern Child Health J.

[19] Odhiambo JN, Sartorius B (2021). Joint spatio-temporal modelling of adverse pregnancy outcomes sharing common risk factors at sub-county level in Kenya, 2016–2019. BMC Public Health.

[20] Liyew AM, Sisay MM, Muche AA (2021). Spatial distribution and factors associated with low birth weight in Ethiopia using data from Ethiopian demographic and health survey 2016: spatial and multilevel analysis. BMJ Paediatr Open.

[21] Kazembe LN, Kandala NB (2015). Estimating areas of common risk in low birth weight and infant mortality in Namibia: A joint spatial analysis at sub-regional level. Spat Spatiotemporal Epidemiol.

[22] Kumari N, Algur K, Chokhandre PK, Salve PS (2021). Low birth weight among tribal in India: evidence from national family health survey-4. Clin Epidemiol Glob Health.

[23] Donal D, Hartono H, Hakimi M, Emilia O (2017). Spatial patterns associating low birth weight with environmental and behavioral factors. Int J Public Health Sci (IJPHS).

[24] WHO. Every newborn: an action plan to end preventable deaths. Geneva: World Health Organization; 2014. https://apps.who.int/iris/handle/10665/127938. Accessed 15 Feb 2023.

[25] WHO. Strategies towards ending preventable maternal mortality (EPMM). Geneva: World Health Organization; 2015. https://www.who.int/publications/i/item/9789241508483. Accessed 16 Jan 2023.

[26] Seale AC, Barsosio HC, Koech AC, Berkley JA (2015). Embedding surveillance into clinical care to detect serious adverse events in pregnancy. Vaccine.

[27] Kamau A, Musau M, Mwakio S, Amadi D, Nyaguara A, Bejon P (2022). Impact of intermittent presumptive treatment for malaria in pregnancy on hospital birth outcomes on the Kenyan coast. Clin Infect Dis.

[28] Scott JAG, Bauni E, Moisi JC, Ojal J, Gatakaa H, Nyundo C (2012). Profile: The Kilifi health and demographic surveillance system (KHDSS). Int J Epidemiol.

[29] Irimu G, Ogero M, Mbevi G, Kariuki C, Gathara D, Akech S (2018). Tackling health professionals’ strikes: An essential part of health system strengthening in Kenya. BMJ Global Health.

[30] Ong’ayo G, Ooko M, Wang’ondu R, Bottomley C, Nyaguara A, Tsofa BK (2019). Effect of strikes by health workers on mortality between 2010 and 2016 in Kilifi, Kenya: a population-based cohort analysis. Lancet Glob Health.

[31] Waithaka D, Kagwanja N, Nzinga J, Tsofa B, Leli H, Mataza C (2020). Prolonged health worker strikes in Kenya- perspectives and experiences of frontline health managers and local communities in Kilifi County. Int J Equity Health.

[32] Chibwesha CJ, Zanolini A, Smid M, Vwalika B, Phiri Kasaro M, Mwanahamuntu M (2016). Predictors and outcomes of low birth weight in Lusaka, Zambia. Int J Gynecol Obstet.

[33] Kamala BA, Mgaya AH, Ngarina MM, Kidanto HL (2018). Predictors of low birth weight and 24-hour perinatal outcomes at Muhimbili national hospital in Dar es Salaam, Tanzania: a five-year retrospective analysis of obstetric records. Pan Afr Med J.

[34] Doctor Hv, Nkhana-Salimu S, Abdulsalam-Anibilowo M (2018). Health facility delivery in sub-Saharan Africa: Successes, challenges, and implications for the 2030 development agenda. BMC Public Health.

[35] Gabrysch S, Nesbitt RC, Schoeps A, Hurt L, Soremekun S, Edmond K (2019). Does facility birth reduce maternal and perinatal mortality in Brong Ahafo, Ghana? A secondary analysis using data on 119 244 pregnancies from two cluster-randomised controlled trials. Lancet Glob Health.

[36] Kruk ME, Chukwuma A, Mbaruku G, Leslie HH (2017). Variation in quality of primary-care services in Kenya, Malawi, Namibia, Rwanda, Senegal, Uganda and the United Republic of Tanzania. Bull World Health Organ..

[37] Guagliardo MF (2004). Spatial accessibility of primary care: concepts, methods and challenges. Int J Health Geogr..

[38] Ouma P, Macharia PM, Okiro, E, Alegana V. Methods of measuring spatial accessibility to health care in Uganda. In: Makanga, P.T. (eds) Practicing Health Geography. Global Perspectives on Health Geography. Springer, Cham; 2021. p. 77–90.

[39] Hyde E, Bonds MH, Ihantamalala FA, Miller AC, Cordier LF, Razafinjato B (2021). Estimating the local spatio-temporal distribution of malaria from routine health information systems in areas of low health care access and reporting. Int J Health Geogr.

[40] Kulldorff M (1997). A spatial scan statistic. Commun Stat Theory Methods.

[41] Kioko EM (2017). Conflict resolution and crime surveillance in Kenya: Local peace committees and Nyumba Kumi. Africa Spectrum.

[42] Macharia PM, Giorgi E, Noor AM, Waqo E, Kiptui R, Okiro EA (2018). Spatio-temporal analysis of Plasmodium falciparum prevalence to understand the past and chart the future of malaria control in Kenya. Malar J.

[43] Macharia PM, Joseph NK, Snow RW, Sartorius B, Okiro EA (2021). The impact of child health interventions and risk factors on child survival in Kenya, 1993–2014: a Bayesian spatio-temporal analysis with counterfactual scenarios. BMC Med.

[44] Mogeni P, Williams TN, Fegan G, Nyundo C, Bauni E, Mwai K (2016). Age, spatial, and temporal variations in hospital admissions with malaria in Kilifi county, Kenya: A 25-year longitudinal observational study. PLoS Med.

