# Supplementary material for: Spatial heterogeneity of low-birthweight deliveries on the Kenyan coast
Source: BMC Pregnancy Childbirth. 2023 Apr 19;23:270. doi: 10.1186/s12884-023-05586-6 (PMC10114419; doi:10.1186/s12884-023-05586-6)
Supplement: Supplementary file 1 — Additional file 1: Fig. S1. The distribution of observed deliveries and weighted under 1 population. The darker the shade the higher the value. Panels A and B: shows the distribution at sub-location level. Panels C and D: shows the distribution at EZ level, respectively. The number of deliveries and weighted under 1 population on the maps are for the entire study period (2011 – 2021). [file 12884_2023_5586_MOESM1_ESM.docx]

**
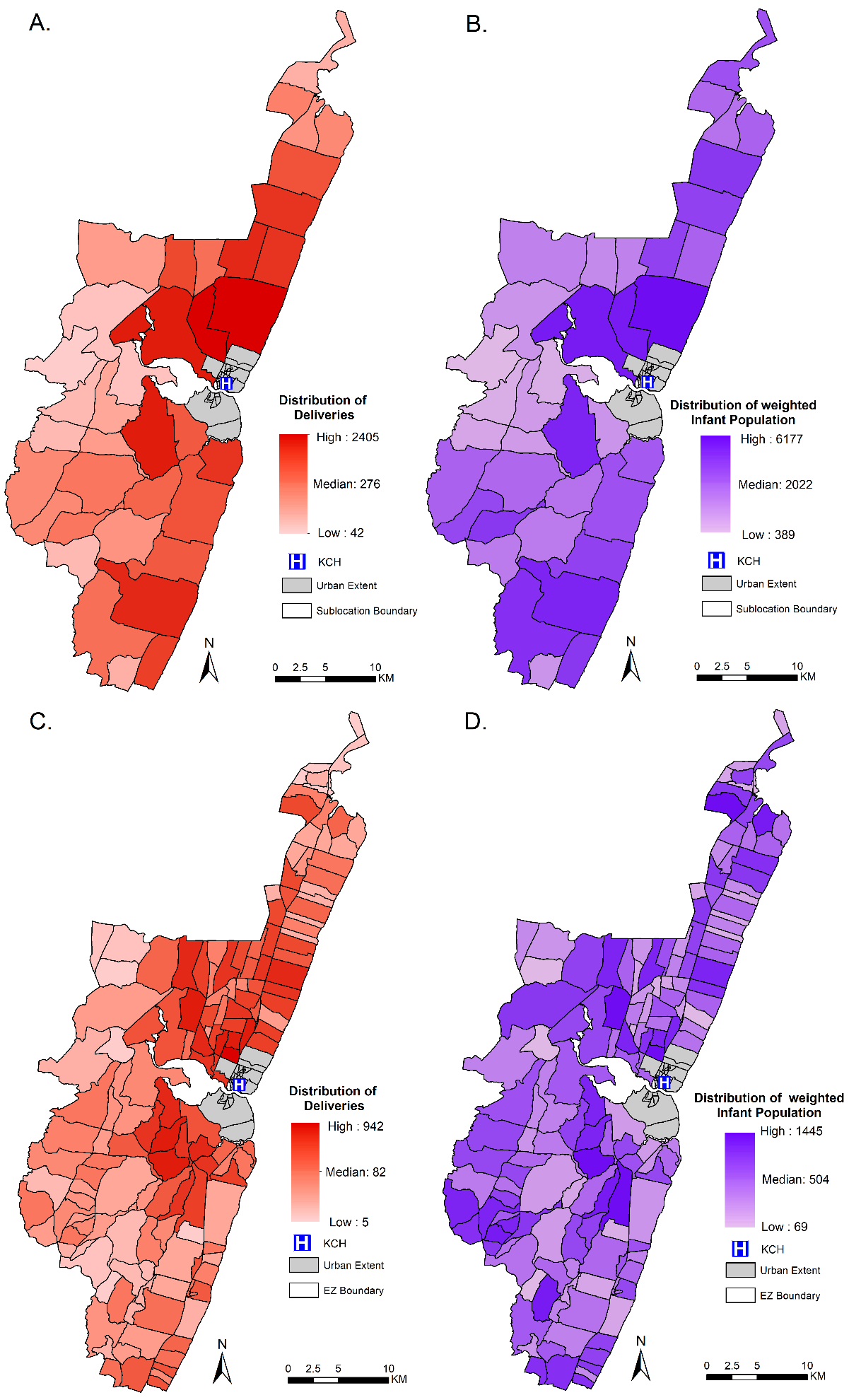
**

**Figure S1:** The distribution of observed deliveries and weighted under 1 population.

The darker the shade the higher the value. **Panels A and B**: shows the distribution at sub-location level. **Panels C and D**: shows the distribution at EZ level, respectively. The number of deliveries and weighted under 1 population on the maps are for the entire study period (2011 – 2021).
