# Supplementary material for: Spatial heterogeneity of low-birthweight deliveries on the Kenyan coast
Source: BMC Pregnancy Childbirth. 2023 Apr 19;23:270. doi: 10.1186/s12884-023-05586-6 (PMC10114419; doi:10.1186/s12884-023-05586-6)
Supplement: Supplementary file 2 — Additional file 2: Table S1. Speeds assigned to different road classes and land covers. Figure S2. Distribution of travel time from EZ centroids to KCH; the darker the shade the longer the travel time. Figure S3. Distribution of access index from EZ centroids to KCH; the darker the shade the higher the access index. [file 12884_2023_5586_MOESM2_ESM.docx]

**Measuring spatial accessibility index**

Access to health services in general is conceptualized in a number of ways. In the broadest sense, there is potential spatial access (proximity to a health facility that one can visit), potential aspatial access (having culturally appropriate care available that one can access), realized spatial access (actually visiting the health facility close by), and realized aspatial access (seeing a health care provider from one’s own cultural group) (1). Data from passive surveillance systems is often characterized as inaccurate and incomplete due to both supply and/or demand associated factors. Although the KIPMAT study improved the accuracy of data at KCH, it was not representative of the utilization levels. Possible contributing factors for lower hospital births in the rural community are linked to 1) differences in the underlying populations within communities which results in varying likelihood of hospital delivery; and 2) hospital-based passive surveillance misses the vast majority of cases in rural settings with low access to hospitals (2–4). For example, less than a third of births in low middle-income countries occur in hospitals as a result of a two-tiered model of care that directs “low risk” women to primary care clinics and “high risk” women to hospitals (4,5). To test for whether there was a bias in the incidence of LBW due to geographic access to health care, we assumed that there was no relationship between observed LBW incidence and access to health care. This criterion was assessed using the Spearman’s rank correlation test, a measure for the strength and direction of the monotonic relationship between LBW incidence and access to health care. To limit bias in the estimation of LBW incidence due to geographic access to KCH, we aimed to adjust for spatial accessibility index. We have focused on the potential spatial access, defined as proximity to KCH.

*Travel time*

The level of geographic access between population residences and health facilities is determined using travel time between these two points (1). As such, travel time from the centroid of each EZ to KCH was estimated using previously published datasets on the impedance surface (6) using the AccessMod 5.6.0 Software. The algorithm in AccessMod models travel time using the terrain-based least cost path distance calculation and is widely applied in the computation of spatial access metrics (7). Briefly, road network, barriers, land cover, travel scenario and digital elevation model datasets were the inputs in AccessMod. Varying travel speeds based on previous studies in the area (8) were assigned to different road classes and land covers (Table S1). Travel time was extracted at the centroids of each EZ from the resulting travel time raster in ArcGIS version 10.5 using the Extract Values to Points function (Figure S2).

**Table S1:** Speeds assigned to different road classes and land covers.

| **Category** | **class** | **label** | **speed (km/hr)** | **mode** |
| --- | --- | --- | --- | --- |
| LandCover | 1 | Tree_Cover | 3.5 | WALKING |
|  | 2 | Shrub_Cover | 4.5 | WALKING |
|  | 3 | Grassland | 4 | WALKING |
|  | 4 | Cropland | 3.5 | WALKING |
|  | 8 | Built_up | 5 | WALKING |
| Roads | 1011 | primary | 80 | MOTORIZED |
|  | 1012 | secondary | 50 | MOTORIZED |
|  | 1013 | minor | 11 | MOTORIZED |
|  | 1016 | rural | 5 | WALKING |


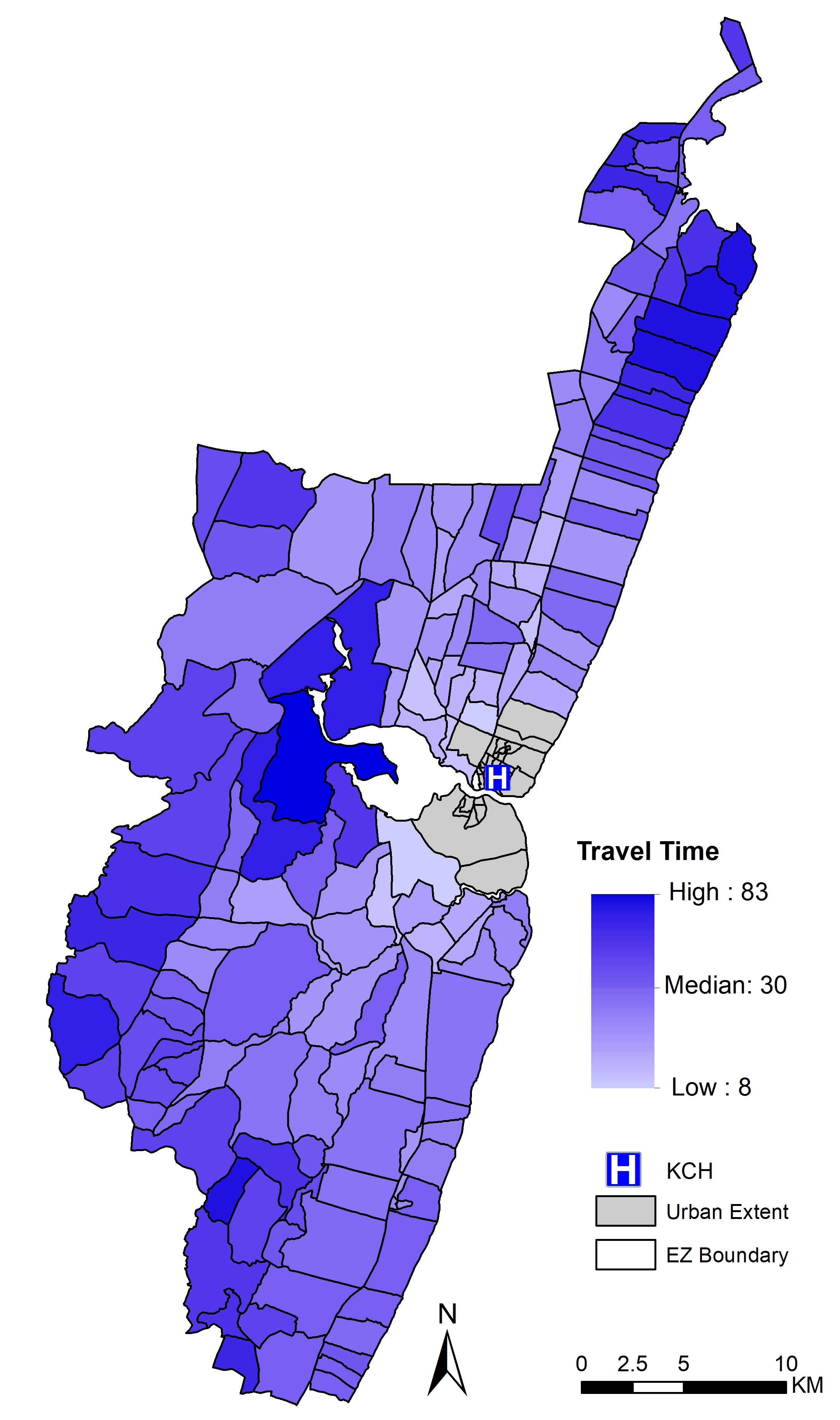


**Figure S2:** Distribution of travel time from EZ centroids to KCH; the darker the shade the longer the travel time.

*Geographic Access Index*

The likelihood of an event occurring at a health facility decreases with increasing distance from the health facility (3,9,10). However, travel impedance on its own is a poor indicator of spatial accessibility. Combined measures of travel impedance (accessibility) and supply (availability) are necessary to properly understand spatial accessibility. Gravity models attempt to represent the potential interaction between populations and service points such that the likelihood of interaction decreases with increasing distance or travel impedance (11). The accessibility index under the gravity model for a region i is the summation of all the available supply adjusted by a function of travel time (Figure S3):

$$A_{i}=\sum_{j} \frac{S_{j}}{{f(tt}_{\mathrm{ij}})}$$

where A_i_ is the accessibility index for EZ_i_, S_j_ is the supply for facility j, for a single health facility (i.e. KCH) S_j_ was set at 1 and f(tt_ij_) is the transformation of travel time between EZ_i_ and KCH_j_ such that:

$$f\left( \mathrm{tt}_{\mathrm{ij}} \right)=\left\{ \begin{aligned} 1, &tt<10 \\ \frac{\mathrm{tt}}{10}, &10\geq tt\geq120 \\ \infty, tt>120 \end{aligned} \right.$$

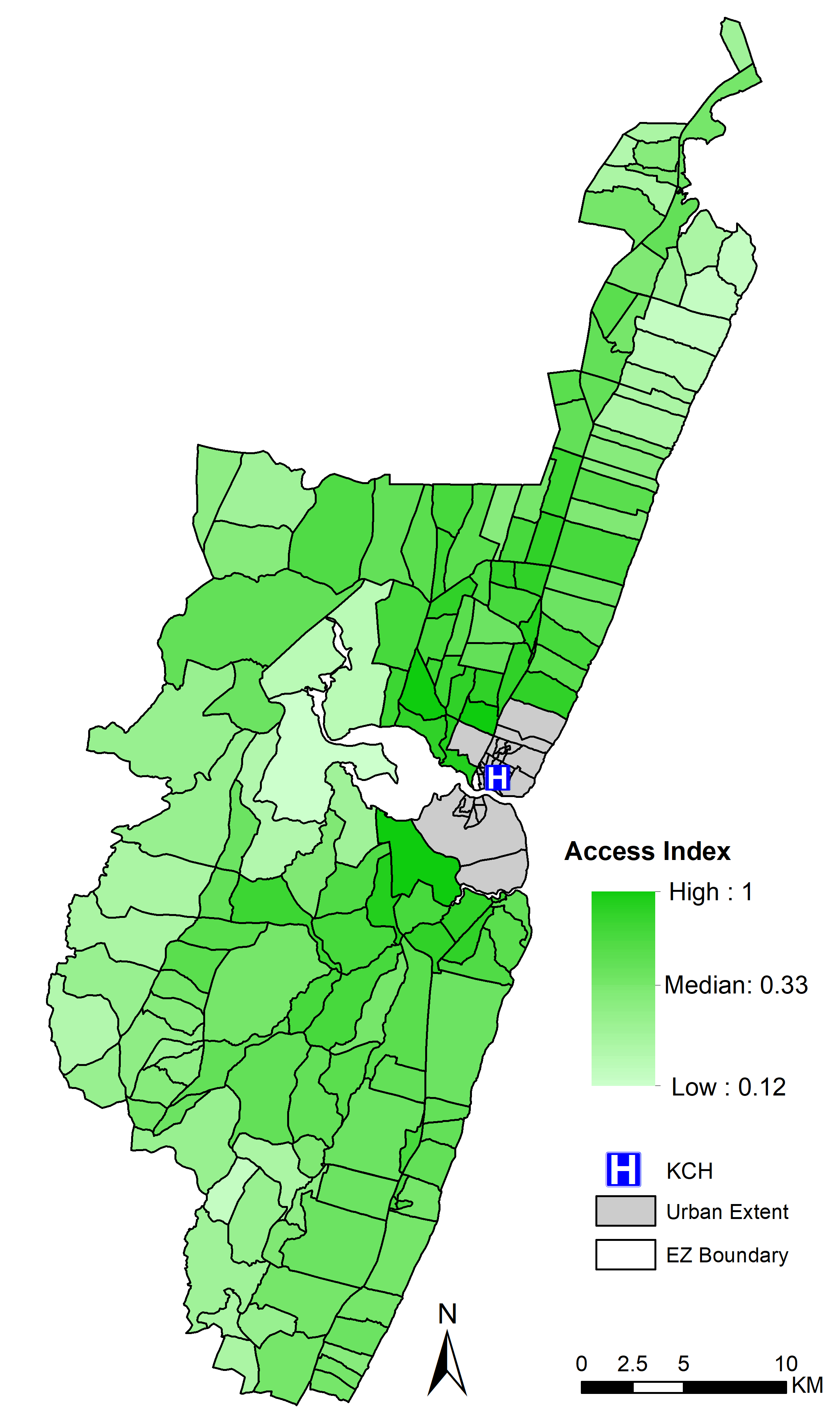


**Figure S3**: Distribution of access index from EZ centroids to KCH; the darker the shade the higher the access index.

**References**

1. Crooks VA, Schuurman N. Interpreting the results of a modified gravity model: Examining access to primary health care physicians in five Canadian provinces and territories. BMC Health Serv Res. 2012;12(1).
2. Gabrysch S, Campbell OMR. Still too far to walk: Literature review of the determinants of delivery service use. BMC Pregnancy Childbirth. 2009;9.
3. Doctor H v., Nkhana-Salimu S, Abdulsalam-Anibilowo M. Health facility delivery in sub-Saharan Africa: Successes, challenges, and implications for the 2030 development agenda. BMC Public Health. 2018 Jun 19;18(1).
4. Gabrysch S, Nesbitt RC, Schoeps A, Hurt L, Soremekun S, Edmond K, et al. Does facility birth reduce maternal and perinatal mortality in Brong Ahafo, Ghana? A secondary analysis using data on 119 244 pregnancies from two cluster-randomised controlled trials. Lancet Glob Health. 2019;7(8).
5. Kruk ME, Chukwuma A, Mbaruku G, Leslie HH. Variation in quality of primary-care services in Kenya, Malawi, Namibia, Rwanda, Senegal, Uganda and the United Republic of Tanzania. Bull World Health Organ. 2017;95(6).
6. Ouma PO, Maina J, Thuranira PN, Macharia PM, Alegana VA, English M, et al. Access to emergency hospital care provided by the public sector in sub-Saharan Africa in 2015: a geocoded inventory and spatial analysis. Lancet Glob Health. 2018 Mar 1;6(3):e342–50.
7. Bouanchaud P, Macharia PM, Demise EG, Nakimuli D. Comparing modelled with self-reported travel time and the used versus the nearest facility: modelling geographic accessibility to family planning outlets in Kenya. BMJ Glob Health. 2022 May;7(5):e008366.
8. Ogero M, Orwa J, Odhiambo R, Agoi F, Lusambili A, Obure J, et al. Pentavalent vaccination in Kenya: coverage and geographical accessibility to health facilities using data from a community demographic and health surveillance system in Kilifi County. BMC Public Health. 2022 Dec 1;22(1).
9. Hyde E, Bonds MH, Ihantamalala FA, Miller AC, Cordier LF, Razafinjato B, et al. Estimating the local spatio‐temporal distribution of malaria from routine health information systems in areas of low health care access and reporting. Int J Health Geogr. 2021 Dec 1;20(1).
10. Zhou G, Afrane YA, Malla S, Githeko AK, Yan G. Active case surveillance, passive case surveillance and asymptomatic malaria parasite screening illustrate different Age distribution, spatial clustering and seasonality in western Kenya. Malar J. 2015;14(1).
11. Guagliardo MF. Spatial accessibility of primary care: concepts, methods and challenges [Internet]. Vol. 3, International Journal of Health Geographics. 2004. Available from: http://www.ij-healthgeographics.com/content/3/1/3
