# Supplementary material for: Spatial heterogeneity of low-birthweight deliveries on the Kenyan coast
Source: BMC Pregnancy Childbirth. 2023 Apr 19;23:270. doi: 10.1186/s12884-023-05586-6 (PMC10114419; doi:10.1186/s12884-023-05586-6)
Supplement: Supplementary file 3 — Additional file 3: Figure S4. The association between LBW incidence and travel time. Panels A and C: shows the distance decay for unadjusted LBW incidence at sub-location and EZ level, respectively. Panels B and D: shows the distance decay for adjusted LBW incidence at sub-location and EZ level, respectively. [file 12884_2023_5586_MOESM3_ESM.docx]

**
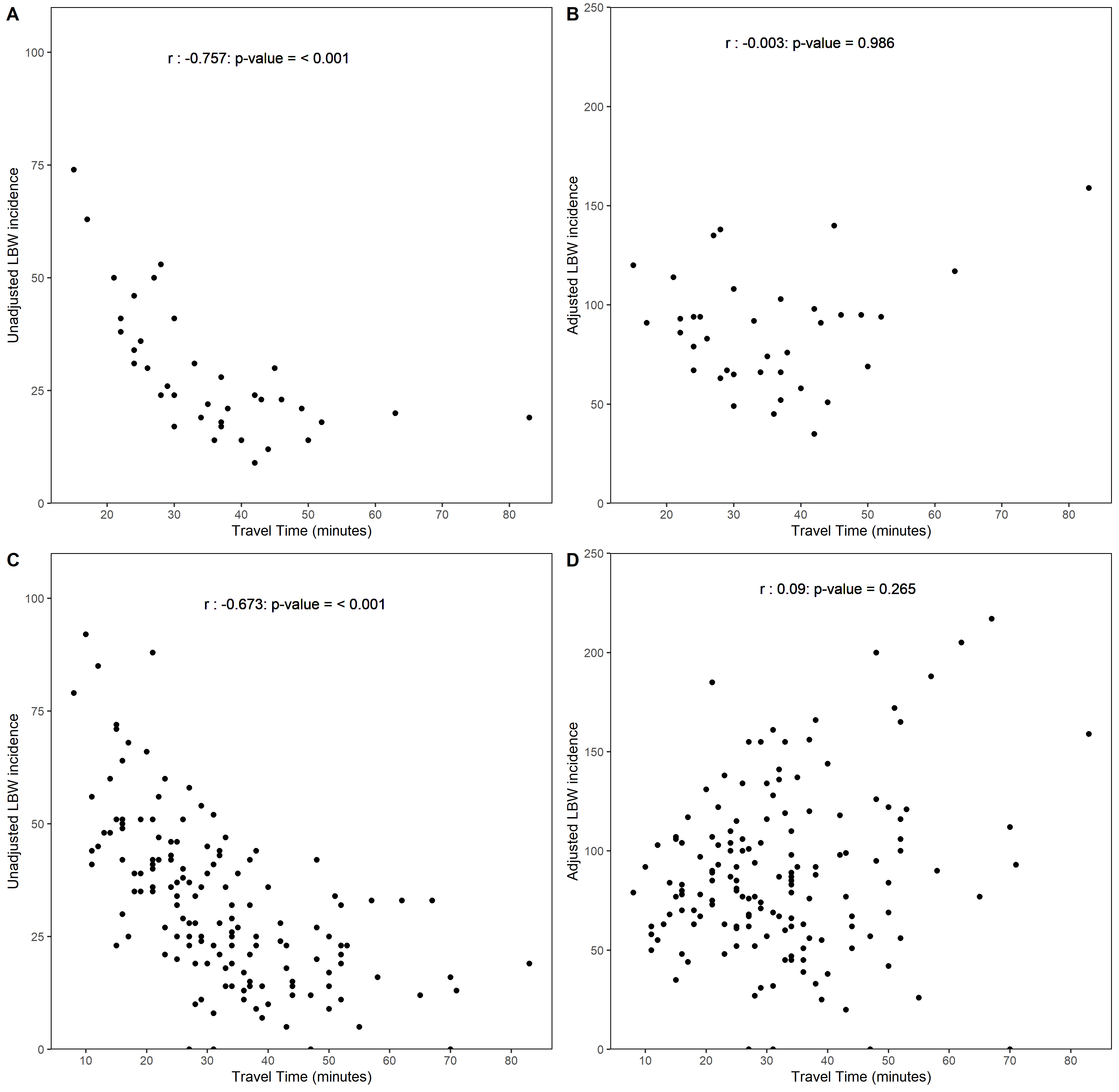
**

**Figure S4:** The association between LBW incidence and travel time. **Panels A and C**: shows the distance decay for unadjusted LBW incidence at sub-location and EZ level, respectively. **Panels B and D**: shows the distance decay for adjusted LBW incidence at sub-location and EZ level, respectively.
