# Supplementary material for: Spatial heterogeneity of low-birthweight deliveries on the Kenyan coast
Source: BMC Pregnancy Childbirth. 2023 Apr 19;23:270. doi: 10.1186/s12884-023-05586-6 (PMC10114419; doi:10.1186/s12884-023-05586-6)
Supplement: Supplementary file 4 — Additional file 4: Figure S5. Sensitivity analysis using varying maximum scanning radius for cluster identification using SaTScan software. Panel A and B: The radius was set at 2 km and 2.3 km at sub-location level, respectively. Panel C and D: The radius was set at 2 km and 2.3 km at EZ level, respectively. The clusters identified were in similar locations as those reported using the 1 km radius for the entire study period (2011 – 2021). [file 12884_2023_5586_MOESM4_ESM.docx]

**
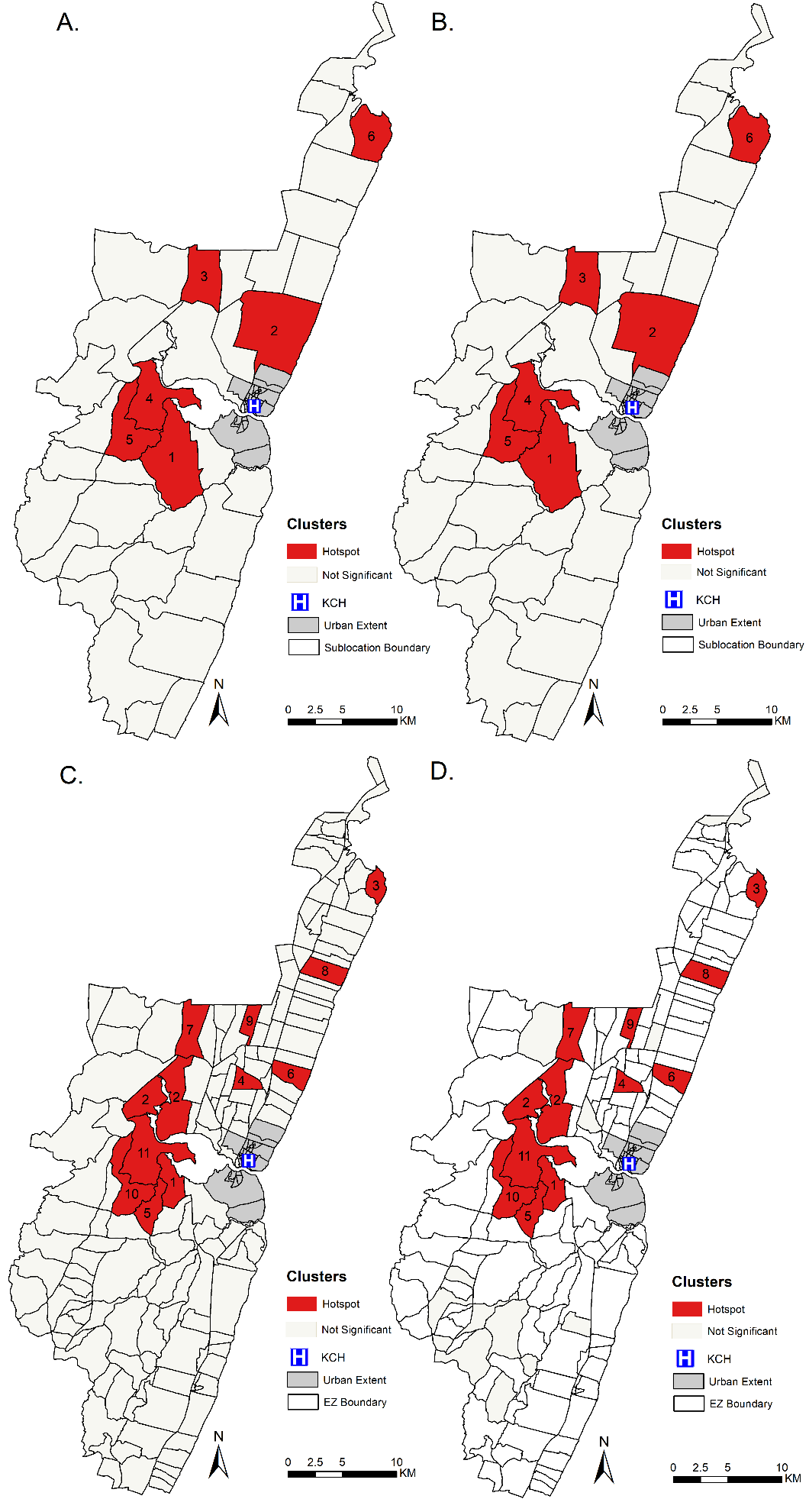
**

**Figure S5:** Sensitivity analysis using varying maximum scanning radius for cluster identification using SaTScan software. **Panel A and B:** The radius was set at 2 km and 2.3 km at sub-location level, respectively. **Panel C and D:** The radius was set at 2 km and 2.3 km at EZ level, respectively. The clusters identified were in similar locations as those reported using the 1 km radius for the entire study period (2011 – 2021).
